# WASH in India: need for innovation

**DOI:** 10.1016/j.lansea.2023.100336

**Published:** 2023-12-05

**Authors:** The Lancet Regional Health – Southeast Asia

The lifetime contributions of Dr Bindeshwar Pathak—a champion of sanitary innovation in India—were eternalised in a moving obituary in *The Lancet* last month. He accomplished the behemoth task of liberating and rehabilitating manual scavengers and preventing disease and death by providing pour-flush toilet technology, which served as an alternative to dry latrines commonly used in India during the 1970s. He is remembered not only as an early reformer in public health and sanitation but also for the social transformation in the lives of millions.

Open defecation is associated with high under-5 mortality, malnourishment, and poverty, with large disparities in socioeconomic groups. There are also gender disparities in access to sanitation and impact of inadequate sanitation. **Women** and girls are at higher risk of disease, anxiety, sexual harassment, rape, and violence due to lack of safe and private sanitation. This proves to be a barrier to **girls' health, education**, and, further empowerment. Poor sanitation is linked to transmission of diarrhoeal diseases such as cholera, typhoid, intestinal worm infections, and polio. In 2020, 44% of the household wastewater generated globally was discharged without safe treatment.

South Asia and Africa have the largest number of people practising open defecation in the world. India, Indonesia, and Nepal have the highest number of people practising open defecation in southeast Asia, primarily due to lack of basic sanitation infrastructure, social norms, and cultural or habitual preferences. Bangladesh has been successful, in reducing open defecation from 34% in 1990 to below 1% in 2016. **Sri Lanka** is another country that wiped out open defecation in 2017. Both these countries have similar cultural issues with sanitation but were able to achieve open defecation-free status with strong political will.

Sustainable Development Goal target 6.2 calls for adequate and equitable sanitation for all, and target 6.3 calls for halving the proportion of untreated wastewater and substantially increasing recycling and safe reuse. The National Annual Rural Sanitation Survey of India reported that 93% of rural households in India had toilets by 2019 as a result of concerted efforts by the government, with 96.5% of people who had access to toilets using them; however, 19 million people still practised open defecation. Behavioural change, maintenance of latrines, and water access issues are cited as obstacles that continued to block India's goal of being 100% open defecation-free. Technology innovations such as bucket toilets, portable toilets, bio-septic tanks, and dry toilets are all in the pipeline; however, acceptability, cost, and sustainability will remain key to adoption. A WHO study in 2012 calculated that for every US$1.00 invested in sanitation, there was a return of US$5.50 in lower health costs, more productivity, and fewer premature deaths. Without this investment, every year 830,000 people will die from preventable diseases.

As a step ahead, India committed to waste management, in its next phase of sanitation goals in addition to further elimination of open defecation. Currently, only 27% of household water is safely treated in India. Wastewater and sludge is now considered a valuable resource for food production; however, if untreated, this is ridden with disease risk. As per the 2011 census, at the country level, there is no drainage facility in 48.9% of households in India, while 33% have only an open drainage system. India has no clear policy on wastewater treatment that is running with limited or poor infrastructure. Big cities with centralised sewage systems are incapable of taking the current population burden and are falling apart during seasonal floods, risking the health and lives of millions. On the other hand, small towns and rural areas that do not have well-developed sewage systems due to their cost and maintenance rely on septic tanks (pit latrines) for collecting sewage, which must be emptied once they are full. These pits are also located very close to drinking water sources and hence pose a very high risk of contamination, leading to seasonal outbreaks of diarrhoeal and other infectious diseases Current crises of climate disasters like water shortage and floods remind us of the rational use of water. However, existing latrines use huge amounts of water that might not be sustainable. We need a drastic and urgent revision of current toilet use in countries most vulnerable to climate change. These countries also do not have the resources to quickly adopt newer or more advanced technology in the face of disasters.

India has built toilets, pulling 500 million additional people out of the practice of open defecation over last 10 years. However, there continues to be the menace of human waste that lies untreated and threatens human health. Adopting water, sanitation, and hygiene (WASH) innovations can also support equality and universality of services, helping extend sanitation to the hardest-to-reach areas and groups. With increasing urbanisation and migration, Pathak's innovation has proved to be a blessing to large populations living in villages and slums in cities. To advance the Sustainable Development Goals and provide safer environments for people, we need advocates like Pathak, who understood the needs of local people and could adapt solutions to such needs that were practical, cost-effective, and scalable.

